# A Hybrid Ligand- and Structure-Based Drug Design Invoked through Square Attention in Protein–Ligand Transformer

**DOI:** 10.1021/acsomega.6c04325

**Published:** 2026-10-08

**Authors:** Yanhui Lu, Yasunori Ohara, Hayato Kunugi, Masaru Tateno

**Affiliations:** Innovation to Implementation Laboratories, Central Pharmaceutical Research Institute, Japan Tobacco Inc., Takatsuki, Osaka 569-1125, Japan

## Abstract

Computer-aided molecular design based on evaluation of protein–ligand (PL) binding affinity is important for accelerating drug discovery. A deep-learning model for natural language processing (NLP) involving an attention mechanism (i.e., Transformer) is expected to improve the PL-affinity evaluation along with clarifying amino acid residues and ligand atoms that are responsible for molecular interactions. However, the original attention (i.e., a scaled dot-product with row-wise Softmax normalization) is an adapted method for relationships between homogeneous inputs (e.g., two sentences in the translation tasks) and thus is not suitable to analyze heterogeneity found in input data such as proteins (10^2^–10^3^ residues) and ligands (10^1^–10^2^ atoms). In fact, due to the significant difference between the numbers of protein residues and ligand atoms, gaps in the scale of attention weights emerge after the normalization, which thus affects model performance. Herein, we report PLTransformer, which involves our novel attention mechanism with Softmax normalization exerting on both directions of protein residues and ligand atoms in a more equivalent manner, for correcting the imbalance by a tandem of attention blocks with swapped inputs. As a result, our PLTransformer shows competitive affinity predictive performance over a representative baseline while also providing the ability to identify binding pockets, including allosteric sites, and important ligand scaffolds and protein substructures without employing three-dimensional (3D) structures of PL complexes (i.e., a ligand-based drug design (LBDD) framework). Thus, the possible PL (residue–ligand atom) contacts provided by PLTransformer can assist us to select plausible PL binding modes obtained by docking simulations, promoting the identification of the appropriate binding mode such as to maximize the performance of PLTransformer (i.e., structure-based drug design (SBDD) framework).

## Graphical abstract

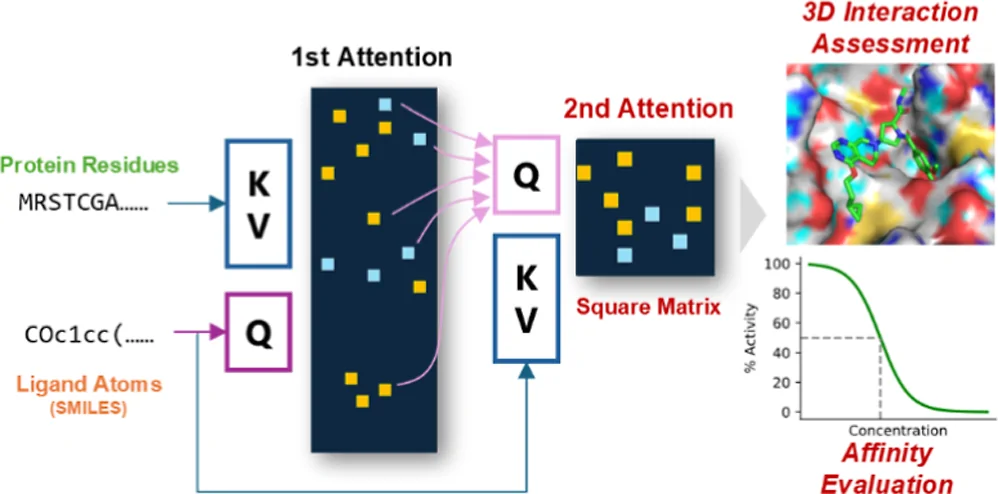

## Introduction

Protein–ligand (PL) binding affinity calculation by docking simulation and molecular dynamics (MD) calculation^1^ has been widely used in modern structure-based drug design (SBDD). Thanks to the success of AlphaFold^2^ and RoseTTAFold,^3^ proteins with unknown 3D structures can now be incorporated into SBDD^4,5^ strategies using predicted structures. Recently, AlphaFold3 ^6^ was reported to have the ability to predict PL (including small chemical compounds, nucleic acids, antibodies, and proteins) complexes with high accuracy. However, not all predicted structures are suitable for SBDD, given that only 36% of all residues have very high confidence levels.^7^ Moreover, proteins with unknown cryptic binding sites or allosteric binding sites still remain difficult due to the varied mechanisms of allosteric effects and high computational costs.^8^ Moreover, the expensive computation that is required by molecular simulations limits the ligand search space, in particular, when we aim at applying these to ultralarge virtual ligand chemical spaces.^9^

Recently, a lot of deep learning-based models have been developed to predict PL binding affinities.^10–13^ Owing to their computational efficiency, these methods are usually used in the early stages of virtual screening with large databases, followed by molecular docking and MD simulations.^14,15^ In more detail, deep learning methods can be classified into two categories based on whether they use three-dimensional (3D) structures of proteins: sequence-based methods and structure-based methods. Sequence-based models are trained using data including PL pairs represented by sequences such as characters of amino acid residues for proteins and SMILES strings for ligands.^16^ In this methodology, PL interactions are implicitly encoded within the latent spaces of their embeddings (ligand-based drug design (LBDD)), which are generated by a neural network that processes their combined representations. On the other hand, structure-based models estimate the binding affinity, explicitly using 3D structures of PL complexes. 3D-geometrical and topological information on PL complexes are utilized in the structure-based models for accurate estimation and the interpretability on the interactions (structure-based drug design (SBDD)). However, the structure-based models are trained on a more limited amount of data; also from a chemical viewpoint, the large proportion of available data sets on PL affinity does not include structural information.

Inspired by the great ability of Transformer^17^ in capturing relationships between two sequences, deep learning approaches using an attention mechanism^18^ have become popular for drug-target binding affinity prediction.^19–37^ During binding affinity analysis, attention matrices are generated as intermediate products, which can be indicative of noncovalent interactions (NCIs) between proteins and ligands. As a result, considerable efforts were devoted to analyzing attention matrices to improve model interpretability. In modeling of PL interaction, the attention mechanism mixes different features such as protein sequences and ligand representations, which is in contrast to natural language processing (NLP). Scaled dot-product attention^15^ is widely adopted: the output is the sum of inputs (values), where the weights are determined by the interaction strength between queries and inputs (keys). The interaction strengths are normalized by row-wise Softmax operation (i.e., independently by each query), called the attention weights. This “one-side attention” mechanism was originally proposed in the Transformer architecture for NLP tasks and has been widely used in various fields. In modeling of PL interaction, the one-side attention mechanism was applied to identify which subsequences in the protein are important for the ligand molecules or vice versa.^20,28,34–38^

On the other hand, a two-side attention mechanism was first proposed by Santos et al.,^39^ for pairwise ranking or classification. They developed an approach named attentive pooling (AP), in which the attention matrix—capturing the embedding similarity of each pair during training—is used to derive the corresponding attention vector as a joint representation for each input pair. The AP mechanism is also effective in matching pairs of inputs with significant length variations. Actually, in 2018, Gao et al.^19^ reported that the same method could be applied for PL binding affinity prediction, and thereby attention matrices were demonstrated to explain PL NCIs.

However, a critical issue in applying the two-side attention mechanism is the normalization problem. In the classical attention mechanism, the attention matrix is normalized with the softmax operator. However, two-sided attention mechanisms should normalize the attention matrix on both row-wise and column-wise directions. Sander et al. developed a simple method named Sinkformer^22^ to make a square attention matrix doubly stochastic with the Sinkhorn algorithm, by changing the direction of the Softmax operation at each training epoch. However, it remains mathematically unclear whether a similar methodology can be effectively applied to rectangular matrices, in particular, for cases of protein sequences (usually hundreds of residues) and ligand atoms (usually dozens of atoms).

Instead of directly normalizing the rectangular attention matrix, Santos et al. and Gao et al.^19,39^ proposed max pooling, in which the matrix is transformed into two representative vectors (i.e., row and column vectors), followed by applying softmax to each of them, respectively. The problem of differing lengths is resolved, however, Li et al.^28^ reported later that this model achieved average AUC and enrichment scores around of 0.5 and 1, respectively, which were close to the scores of random predictions. Thus, alternative methodologies are required to apply both-sided normalization without reducing dimensions. In this respect, HyperAttentionDTI,^21^ DeepCDA,^36^ and AttentionDTA^40^ operated Sigmoid functions to the attention matrix. Unlike softmax, outputs of the sigmoid function are not normalized and may obscure the relative importance of different elements within the matrix. Thus, the dimension issue (i.e., a significant difference found in the lengths of protein and ligand representation vectors that are used for the inputs of the transformer) when operating softmax normalization remains a serious challenge to be addressed.

Herein, we report a novel two-side attention mechanism, which leads to a fully normalized attention matrix in the row and column directions corresponding to protein sequences (amino acid residues) and ligand atoms (SMILES), respectively, as inputs for PL affinity prediction (our system is referred to herein as PLTransformer). We employed a tandem architecture consisting of two sequential attention blocks: first attention block calculated PL interactions and output vectors whose length equals the number of ligand atoms as “compressed protein representations” and second attention calculated interactions between outputs from first attention and ligand representations. Through the first attention block, the protein representations are summarized so that their length equals the number of ligand atoms; therefore, we can compute square attention between proteins and ligands in the second attention block. Not only is PLTransformer able to predict PL affinities, but it also provides interpretable contact maps of PL interaction through the learned attention matrices. These contact maps enable qualitative investigation of interaction between protein residues and ligand atoms and demonstrate that our model can identify NCI-forming regions in PL complexes. Compared to previous unsupervised models, our model shows significantly improved explainability of PL contacts. Further analysis confirms that our PLTransformer can also identify allosteric sites.

## Methods

### Overview of Model Architecture

PLTransformer is an end-to-end model that takes protein sequences and ligand SMILES as inputs to predict binding affinities (Figure 1). Simultaneously, the model generates attention matrices (protein residues and ligand atoms) as auxiliary products, which provide extensive information about PL NCIs as well as ligand binding sites.

**Figure 1:**
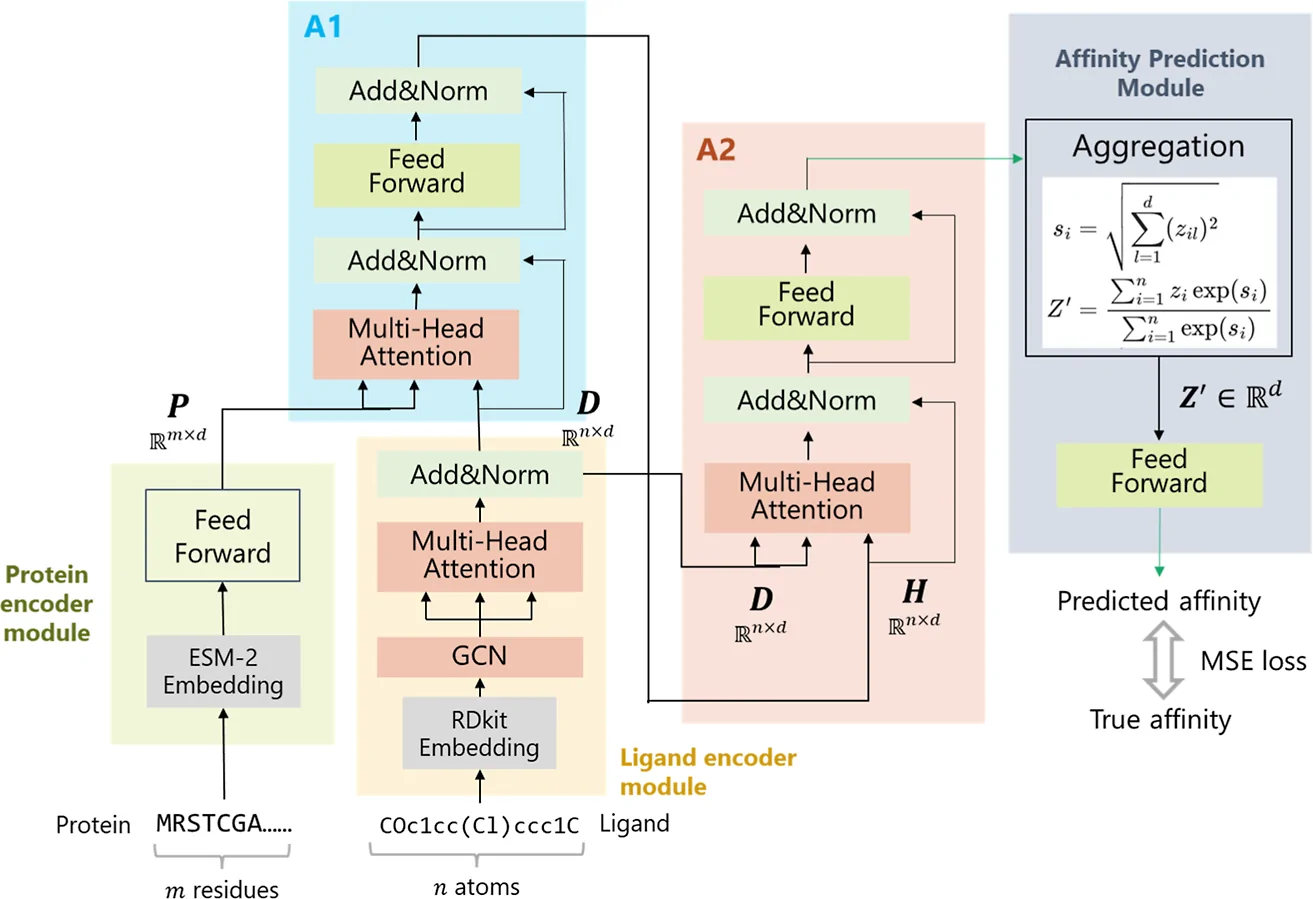
Overview of PLTransformer. Protein sequences are first embedded into 1280-dimensional representations using a pretrained transformer protein language model (ESM-2), and then transformed into protein features *P* through sequence-wise feed forward networks. Ligand atoms are featured by RDKit and further embedded using Graph Convolutional Networks (GCNs). Finally, *n* atom-wise *d*-dimensional representations *D* are obtained after processing by the GCN layers. We here adapt the multihead self-attention layer into a multihead cross-attention layer to learn relationships between protein sequences and ligand atoms (A1). Outputs of A1 are passed to the second attention block (A2) and interactions with ligand atoms are computed. As a consequence, a square attention-weight matrix is constructed. Finally, representations from all atoms are aggregated and the binding affinities are evaluated.

The PLTransformer model comprises a “Protein Encoder Module,” a “Ligand Encoder Module,” two transformer decoders (A1 and A2), and an “Affinity Prediction Module.” The first two modules embed input features into residue-wise and atom-wise representations. The first decoder focuses on extracting important residue features (protein latent features) based on their attention weights given by atom-wise ligand features. The second decoder then takes the protein latent feature and ligand atom-wise features as inputs to interpret PL interactions and produces a complex representation. Finally, the complex representations are aggregated into a single representation, which is then fed into the “Affinity Prediction Module” to predict the binding affinity.

### Protein Embedding Module

In the Protein Encoder module, protein sequences with *m* residues were first embedded into an *m* × *d*_*ESM*2_ matrix representation using a pretrained transformer protein language model, Evolutionary Scale Modeling 2 (ESM-2).^42^ We used the *esm2_t33_650M_UR500* model, which has an embedding dimension of *d*_*ESM*2_ = 1280. ESM-2 features have been utilized for protein structure and function prediction^43,44^ and have already been reported to be useful for drug-target interaction (DTI) prediction.^23,24,33^ The ESM-2 embeddings were further transformed into a *d*-dimensional protein feature $P\in R^{m\times d}$ through sequence-wise feed forward networks.

### Ligand Encoder Module

In the Ligand Encoder Module, each ligand atom was initially represented as a 35-dimensional feature vector using the RDKit python package, and the list of atom features is summarized in Table 1. We then used Graph Convolutional Networks (GCNs) to learn atom representations by integrating features from neighboring atoms, as described by Chen et al.^20,24^ Finally, after processing through the GCN layers, we obtained atom-wise *d*-dimensional representations $D\in R^{n\times d}$ where *n* denotes the number of atoms.

**Table 1:** List of Compound Atom Features

atom type	H, C, N, P, O, S, F, Cl, Br, I or others (one-hot)
degree of atom	0, 1, 2, 3, 4, 5 or 6 (one-hot)
formal charge	0 or 1
number of radical electrons	0 or 1
hybridization type	sp, sp2, sp3, sp3d, sp3d2 or other (one-hot)
number of atoms attached	0, 1, 2, 3 or 4 (one-hot)
chirality	0 (False) or 1 (True)

### Decoders 1 and 2

We adopted Transformer’s encoder architecture,^17^ containing a stack of *n = 6* identical layers, including attention layers, position-wise fully connected feed-forward network, residual connections, and layer normalization. We here adapt the multihead self-attention layer to a multihead cross-attention layer to learn relationships between two modalities.

The first encoder takes *D* from the ligand encoder module as queries and *P* from the protein encoder module as keys and values. Let $A\in R^{n\times m}$ be the calculated attention weights between the query and key linear projections of *D* and *P*, respectively. For each *i*-th atom (*i*∈{1,2,···,*n*}), the attention weights are distributed across all *m* corresponding chemical substructures, where ∑_*j*=1_^*m*^ *A*_*ij*_^1^ = 1 is imposed through a row-wise Softmax normalization, as follows.

H=LayerNorm(D+f(MultiAttn(D,P,P)))

H=LayerNorm(H+FF(H))

where $H\in R^{n\times d}$ is a set of *updated* features with the same size as input query features *D*, *LayerNorm* is a layer-wise normalization method,^45^ *f* indicates the Linear function, *FF* denotes a row-wise feedforward layer, and *MultiAttn* denotes a *k* headed attention layer.

Attention coefficient $A^{1}\in R^{n\times m}$ in every head is defined as follows

$$A^{1}={\left(A_{ij}^{1}\right)}_{i=1,\cdot \cdot \cdot ,n;j=1,\cdot \cdot \cdot ,m},,A_{ij}^{1}=\frac{\exp\left(D_{i}P_{j}^{T}/\sqrt{d_{h}}\right)}{\sum_{j=1}^{m}\exp\left(D_{i}P_{j}^{T}/\sqrt{d_{h}}\right)}$$

On the other hand, the second decoder takes $H\in R^{n\times d}$ from the first Decoder as queries and $D\in R^{n\times d}$ from a ligand encoder module as keys and values. As a result, a square attention-weight matrix is constructed; that is, let $A^{2}\in R^{n\times n}$ be the calculated attention weights between the query and key linear projections of *H* and *D*, respectively, where ∑_*i*=1_^*n*^ *A*_*ki*_^2^ = 1 is adopted through a row-wise Softmax normalization. Thus, it follows

Z=LayerNorm(H+f(MultiAttn(H,D,D))

Z=LayerNorm(Z+FF(Z))

where attention coefficient $A^{2}\in R^{n\times n}$ in every head is defined as follows

$$A^{2}={\left(A_{ki}^{2}\right)}_{k=1,\cdot \cdot \cdot ,n;i=1,\cdot \cdot \cdot ,n},A_{ki}^{2}=\frac{\exp\left(H_{k}D_{i}^{T}/\sqrt{d_{h}}\right)}{\sum_{i=1}^{n}\exp\left(H_{k}D_{i}^{T}/\sqrt{d_{h}}\right)}$$

### Affinity Prediction Module

First, latent features $Z\in R^{n\times d}=\left(z_{1},z_{2},...,z_{n}\right)$ from the second decoder is normalized and aggregated to a vector $Z^{\prime }\in R^{d}$ as described by TransformerCPI.^20^ Then, the affinity prediction module takes *Z*′ as the input to predict the binding affinity score ŷ. Thus, it follows

$$s_{i}=\sqrt{\sum_{l=1}^{d}{\left(z_{il}\right)}^{2}}$$

$$Z^{\prime }=\frac{\sum_{i=1}^{n}z_{i}\exp\left(s_{i}\right)}{\sum_{i=1}^{n}\exp\left(s_{i}\right)}$$

$$ŷ=MLP\left(Z^{\prime }\right)$$

MLP is a two-layered neural network, in which Dropout is applied and ReLU is used as the nonlinear activation function. During training, the loss was calculated based on root mean squared error (RMSE) between predicted binding affinities and the correct labels. The batch-wise loss objective is expressed as follows

$$L\left(Ŷ,Y\right)=\sqrt{\frac{1}{B}\sum_{b=1}^{B}{\left(y_{b}-\hat{y_{b}\;}\right)}^{2}}$$

where *b* = 1, 2,···,*B* is a batch index, *B* is the minibatch size, and $Y={\left\{y_{b}\right\}}_{b=1}^{B}$ is the true affinity.

### Analysis on Attention Weights

From the learned attention matrix, one can obtain the predicted relationship between each residue and atom pair, which may correspond to NCIs between proteins and ligands. We construct the interactive attention matrix between *m* residues of protein and *n* atoms of ligand, denoted by *M*, using the attention matrix from the first decoder $A^{1}\in R^{n\times m}$ and from the second decoder $A^{2}\in R^{n\times n}$ as follows

$$M={\left(A^{1}\right)}^{T}A^{2}$$

To identify the important residues or atoms for the molecular interaction, the rows or columns of the attention matrix are summed; $P_{contact}=\sum_{j=1}^{n}M_{ij}$ for residues and $D_{contact}=\sum_{i=1}^{m}M_{ij}$ for atoms. For the contribution of each atom in the ligand to a specific residue *i*, the attention weights for ligand atoms $\mu \left(i\right)={\left\{\mu {\left(i\right)}_{j}\right\}}_{j=1,\cdot \cdot \cdot n}\in R^{n}$ are calculated by $\mu {\left(i\right)}_{j}={\left(A^{1}\right)}_{ji}\cdot \sum_{k=1}^{n}{\left(A^{2}\right)}_{kj}$ . To assess the interpretability of the entire model, we defined a contact score for the model $S_{contact}\in R$

$$S_{contact}=\frac{1}{m}\sum_{i\in X}{\left(P_{contact}\right)}_{i}$$

where *X* denotes the subset of protein residues included in the true interaction.

### Details of Data Set and Training

The PDBbind data set^41^ (v2020) was used in this study for model comparison. The CASF-2013 ^46^ and CASF-2016 ^47^ benchmark data sets were combined and used as the test set for PDBbind, and others were randomly divided into training and validation sets (10:1, respectively). The main purpose of using the structure-annotated PDBbind data set is to compare the predicted contact maps (attention matrix) with the known correct noncovalent labels to show the ability of our models in predicting PL contacts. Furthermore, our method was used for a specific case study of serine proteinase. As the training end point, we predicted IC50, which is one of the metrics for the binding affinity available from the PDBbind data set. To be precise, IC50 is not a direct indicator of the binding affinity, which is defined as the inhibition coefficient of the biological activity of the target protein. However, IC50 is widely adopted for practical purposes in drug discovery work.

To confirm the abilities for PL affinity prediction and NCI prediction, we compared our model with a one-sided attention mechanism model, TransformerCPI,^20^ with a PDBbind (v2020) data set. For this aim, we herein used the same inputs for proteins and ligands as described in the report of TransformerCPI^20^ and ran a fully hyperparameter optimization for a fair comparison of both models.

To create a serine protease-specific data set, we first obtained a list of proteins belonging to the serine proteinase families from the peptidase database, MEROPS.^61^ These proteins were then used as queries to retrieve PL complex IC50 data from BindingDB.^62^ Negative binders, defined as PL pairs without significant binding affinity, were collected from PubChem^63^ and the pairs with IC50 values greater than 10 μM were selected. To enhance the robust discrimination between positive and negative PL pairs, the affinity values from all negative samples were uniformly assigned to 10 mM. The data sets were randomly divided into training, validation and test sets by 8:1:1 ratio (details are provided in the Supporting Information).

For model training, all IC50 values were subsequently transformed into logarithmic values (pIC50).

## Results and Discussion

### Evaluation of Binding Affinity with and without 3d Structural Information

One purpose of using the PDBbind data set is to evaluate the ability of computational models to predict the PL NCIs as well as affinities, both of which are essential for practical drug design. As shown in Table 3, our model showed better predictive performance than TransformerCPI (the affinity values ranged from 0.40 to 15.22). This improvement could be due to our consideration of the bidirectional attention (i.e., from the protein to ligand and from the ligand to protein) as well as to appropriate normalization enabled by the square attention matrix.

Moreover, in this report, we further perform docking simulations of PL complexes, thereby combining structural information with data obtained from the PLTransformer. Notably, we did not employ our own advanced docking simulation scheme in the present report, which was previously proposed to adopt an advanced framework coupled with quantum electronic structure calculations of PL complexes.^51^ Thus, we investigate whether PLTransformer (an LBDD approach) can improve data obtained by even a conventional docking calculation scheme (an SBDD approach) through the unification of such LBDD and SBDD analyses. In the following sections, we report our case studies of employing our PLTransformer scheme, as described in the Methods section.

### Evaluation of Inference on the Seen Protein and Unseen Ligand Binding Complexes

As a case study, we performed an analysis of beta-secretase 1 (Uniprot id: P56817),^47,52^ as an input protein, which was included in both the training and test data sets, while the analyzed ligands were observed only in the test data set. We identified amino acid residues exhibiting high attention weight values in our present model as possibly important residues for PL interactions (referred to herein as residue clusters) and compared them with the crystal structure of the PL complex (PDBid: 4gid).^52^

As a result of the analysis (Figure 2), all the significant residue clusters that were identified by our PLTransformer model were located around the ligand binding site, with some residues making direct contact with ligand atoms (Figure 2A and C). For analysis of ligand atoms, our PLTransformer identified substructures like sulfonamide, hydroxyamide, and amides (marked in red in the chemical structure depicted in Figure 2B) as important ligand atoms, which actually form hydrogen bonds with proteins in the crystal structure (Figure 2B and C).

**Figure 2:**
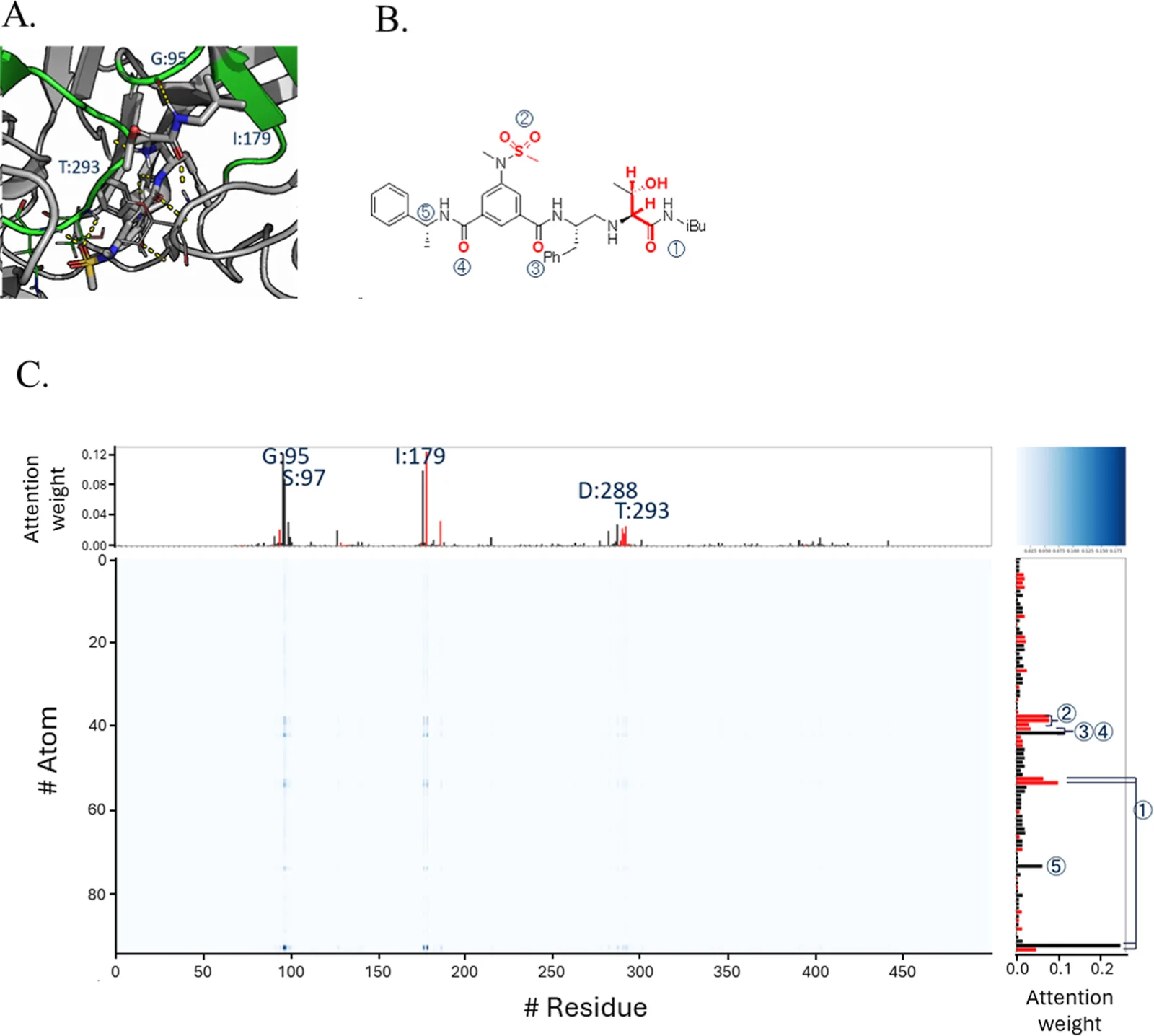
Analysis of beta-secretase 1 (Uniprot id: P56817), which is investigated as a seen protein with an unseen ligand. (**A**) Crystal structure of the PL complex (4gid). Residue clusters identified to be important by PLTransformer are marked in green. Notably, residue clusters are defined as the ones with the highest values and their two neighboring residues. (**B**) 2D structure of ligand in the crystal structure (4gid). Substructures identified to be important by *D*_contact_ PLTransformer are marked in red. (**C**) The top vertical bar chart displays the sum of all attention weights distributed from each residue to all ligand atoms (*P*_contact_), where the red bars represent the ground-truth residues in contact with the ligand. Thus, all residue clusters identified include residues that are located in the ligand-binding site found in the crystal structure. The horizontal bar chart on the right side shows the sum of all attention weights distributed from each atom to all residues (*D*_contact_), where the regions numbered 1–5 correspond to the numbered substructures in (**B**), and the red bars represent the ground-truth atoms in contact with the protein. Thus, atom clusters identified fundamentally include atoms in contact with the protein in the crystal structure (except for substructure 5). The middle heatmap shows the calculated contact matrix (*M*_contact_) and darker colors indicate higher values.

Moreover, we analyzed PL NCIs (Figure 2A), and thus some substructures of ligands were identified to be important, as follows. In the X-ray structure (4gid), a substructure hydroxyamide (1 in Figure 2B) forms hydrogen bonds with residue clusters around G95, and substructures 2–4 (Figure 2B) form hydrogen bonds with a residue cluster around T293. We generated the heatmap of contact matrix obtained by PLTransformer (i.e., contact map) (Figure 2C), which thus shows that the aforementioned PL interactions found in the crystal structure are all involved in the contacts between protein residues and ligand atoms that are identified to be important in the contact matrix (as depicted by cross points in Figure 2C). Notably, significant columns in the contact map are apparently similar to each other as vertical patterns, but actually it is not the case, as discussed later (see Figure S2 in the Supporting Information).

The contact map of PL interaction thus suggests that the substructures 1–5 of the ligand possibly make contacts with all the three residue clusters, rather than each-by-each contact between residues and substructures. Although this may be a feature that is apparently different from those observed in the crystal structure, spatially adjacent moieties, at least, could mutually be influenced in 3D structures, and thus, such an aspect could also be involved in the contact map. To evaluate this issue, we performed molecular docking simulation of the PL complex employing GNINA.^53^ As a consequence, the best pose obtained by the GNINA score function was equivalent to that of the known crystal structure. This result suggests that combining an LBDD approach (PLTransformer) with an SBDD approach (atomistic docking simulation) can be effective to maximize the performance in use of our deep-learning model toward further SBDD works, which enables us to definitely identify the appropriate binding mode, while the contact map provided by PLTransformer may represent an ensemble of PL contacts of possible binding modes.

In this manner, we can further conduct a rational molecular design of ligand via an SBDD approach employing hybrid methods such as contact maps (PLTransformer) and docking examinations (3D structures of PL complexes), which follows highly fast screening of compounds via an LBDD approach (PLTransformer) in a concerted manner (discussed again later).

### Unseen Complexes

For the second case study, we conducted an analysis of Chitinase-3-like protein 1 (UniProt ID: P36222) employing a newly reported structure of the PL complex (8r4x).^54^ As shown in Figure 3A and C, all the important residues observed in the crystal structure were successfully identified by our PLTransformer, including residues that form hydrogen bonds (Y206 and D207) and hydrophobic interactions (W99, F261, and W352). For the ligand, PLTransformer identified a 2,5-substituted morpholine scaffold (1 in Figure 3B: its N atom forms a hydrogen bond with D207 and 5-methyl group blocks the rotation of W99), pyridine moiety (3 in Figure 3B: forming a hydrogen bond with Y206), and the halogen moieties (2 and 4 in Figure 3B: forming hydrophobic interactions). Thus, the present PLTransformer model appropriately recognizes both the protein residue and ligand atoms that are responsible for the PL contacts.

**Figure 3:**
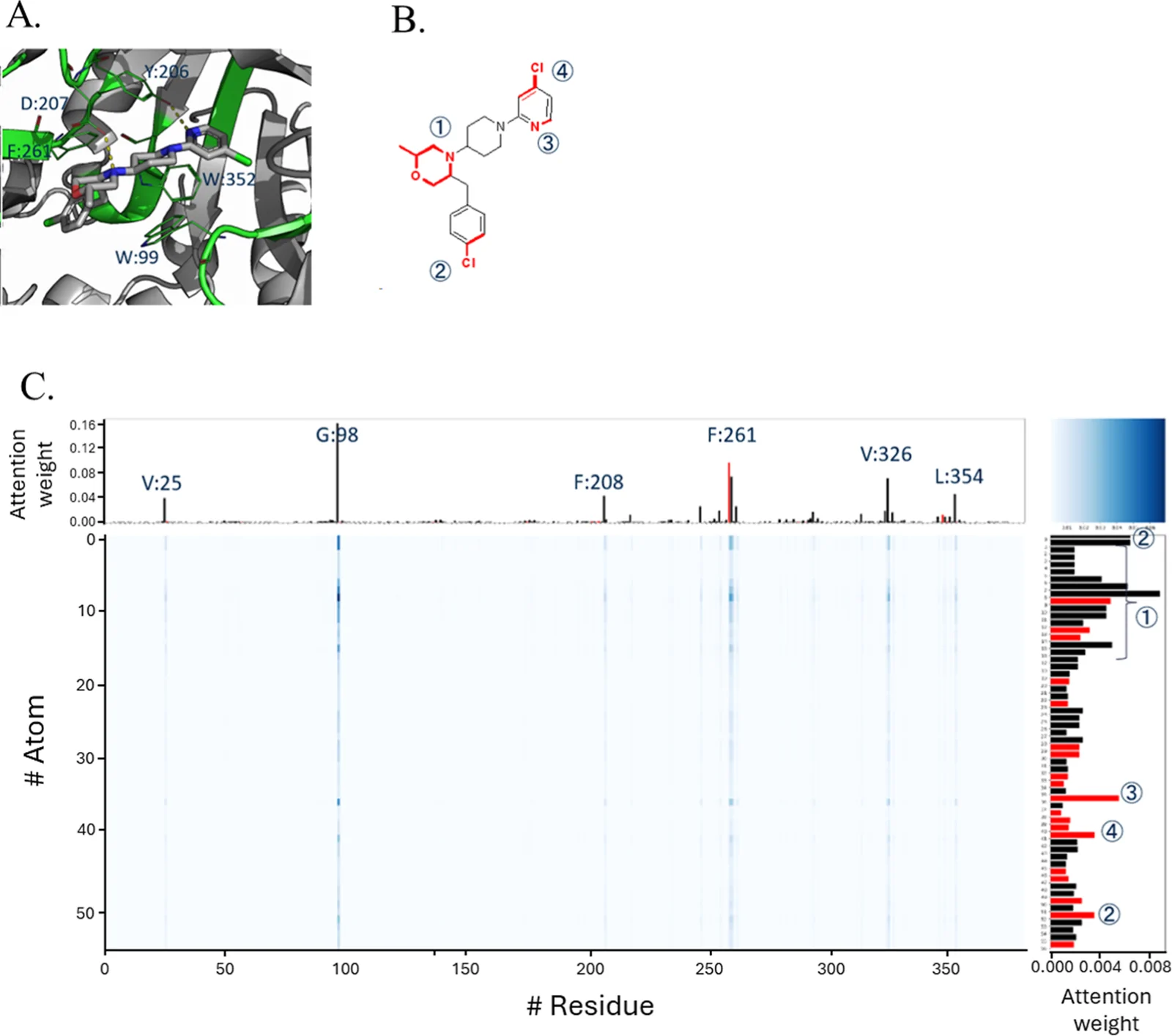
Analysis of Chitinase-3-like protein 1 (UniProt ID: P36222), which is investigated as an unseen complex herein. (**A**) Crystal structure of the PL complex (8r4x). Residue clusters identified to be important by PLTransformer are marked in green. (**B**) 2D structure of ligand in the crystal structure (8r4x). Substructures identified to be important by PLTransformer are marked in red. (**C**) The top vertical bar chart displays the sum of all attention weights distributed from each residue to all ligand atoms (*P*_contact_), where the red bars represent the ground-truth residues in contact with the ligand. The horizontal bar chart on the right side shows the sum of all attention weights distributed from each atom to all residues (*D*_contact_), where the regions numbered 1–4 correspond to the numbered substructures in (**B**), and the red bars represent the ground-truth atoms in contact with the protein. The middle heatmap shows the calculated contact matrix (*M*_contact_), and darker colors indicate higher values.

For analysis of the PL contacts, PLTransformer provides multiple possible binding modes in the contact map; however, specific hydrogen bonds are limited to Y206, D207, and polar ligand atoms (N or O). Consequently, binding modes lacking these key interactions can be excluded. To evaluate this capability in molecular interaction analysis, we performed docking simulation and found that the best pose according to the GNINA score function was actually equivalent to the binding mode observed in the crystal structure. This result demonstrated that combining PLTransformer with docking simulations—used to constrain the structural possibilities—can yield a reasonable binding mode in practical (i.e., blind) molecular design work, following high-performance compound screening using PLTransformer.

### Allosteric Binding Sites

As the third case study, we analyzed the androgen receptor (AR) as the input protein. Several FDA-approved antiandrogens efficiently block endogenous androgen binding at the androgen binding site (ABS) of AR and are widely used in prostate cancer treatment. However, a substantial proportion of patients develop drug resistance, which is mainly caused by mutations in the ABS.^55,56^ In 2007, Estebanez-Perpiná et al.^57^ identified an additional regulatory surface adjacent to the activation function 2 (AF2) cavity, termed binding function 3 (BF3). We used the reported crystal structure (PDBID: 3zqt) as a reference for residues at the BF3 site (Figure 4A). The PDBbind (v2020) training data set contains 19 ligands binding to the ABS and four weak binders to the BF3 site. We examined compound example 153 from WO2023287704A1 as a test ligand for AR using our PLTransformer model. Since no complex structure between compound example 153 and the BF3 site has been reported, we examined a docking simulation using GNINA to model the PL complex structure. In this docking analysis, GNINA successfully generated a plausible binding mode of this ligand at the BF3 site (Figure 4B). Given that GNINA simulation results were consistent with the binding modes observed in known crystal structures in our previous evaluations regarding beta-secretase 1 and Chitinase-3-like protein 1, the binding mode of compound example 153 at the BF3 site shown in Figure 4B is therefore considered to be consistent with the actual binding mode.

**Figure 4:**
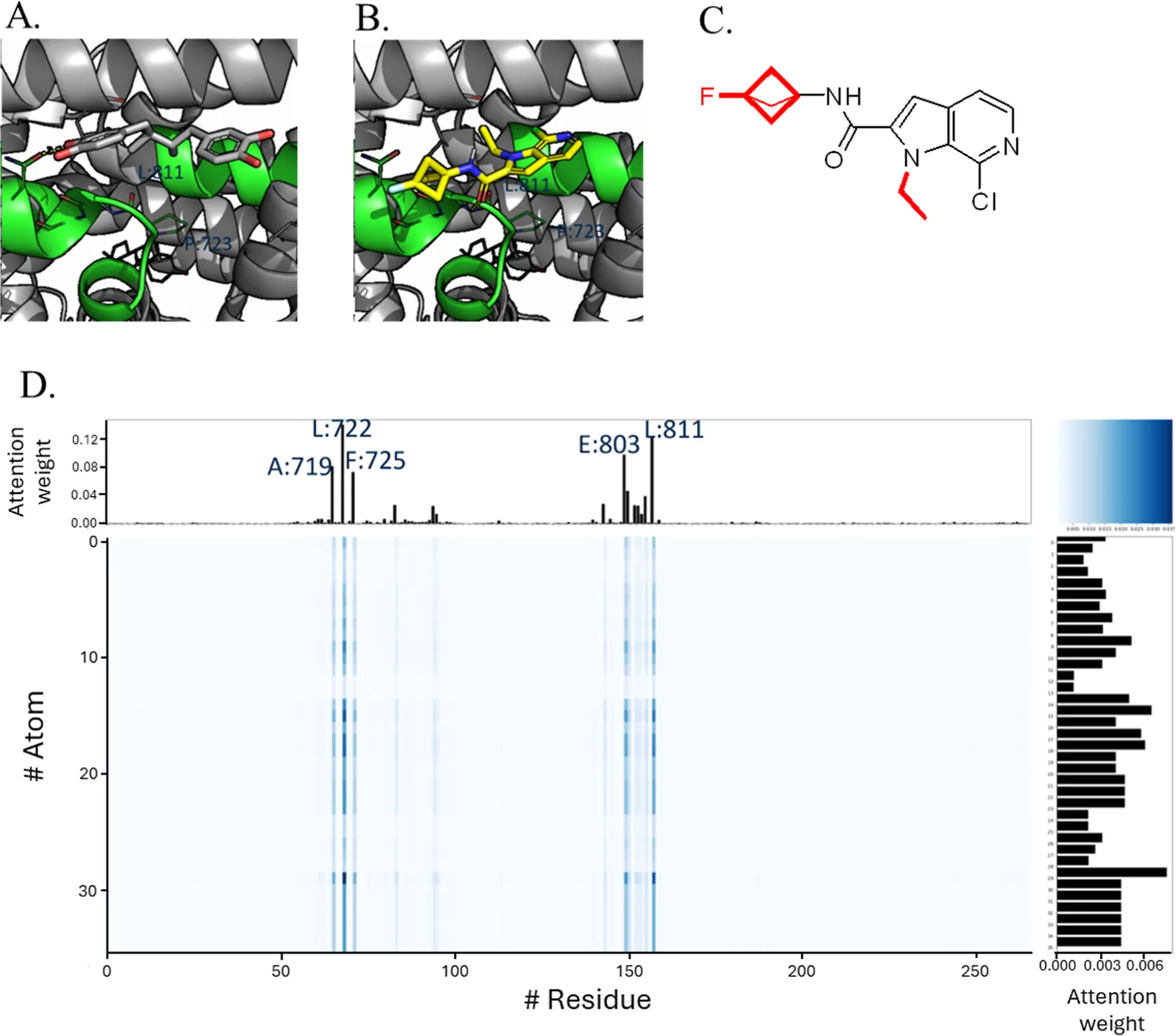
Analysis of the androgen receptor (AR), which is investigated as a seen protein and unseen allosteric ligands. (**A**) Crystal structure of the PL complex (3zqt). Residue clusters identified to be important by PLTransformer are marked in green. (**B**) A docking pose of AR and an allosteric ligand (example 153 in WO2023287704A1) provided by GNINA. (**C**) 2D structure of the ligand shown in (**B**). Substructures identified to be important by PLTransformer are marked in red. (**D**) The top vertical bar chart displays the sum of all attention weights distributed from each residue to all ligand atoms (*P*_contact_), where the red bars represent the ground-truth residues in contact with the ligand. The horizontal bar chart on the right side shows the sum of all attention weights distributed from each atom to all residues (*D*_contact_). The middle heatmap shows the calculated contact matrix (*M*_contact_), and darker colors indicate higher values.

We mapped the attention peaks obtained from PLTransformer by rendering the 3D structures of residues on the crystal structure with BF3-site binders (Figure 4A and B). This analysis revealed that PLTransformer successfully identified protein residues surrounding the allosteric BF3 site as well as key ligand substructures (Figure 4C). Notably, some of these residues were reported to play important roles in allosteric signaling.^58^ In contrast, relatively little attention was assigned to the ligand-binding site (ABS), enabling unambiguous identification of the binding site (Figure 4D). This behavior may reflect an advantage of our PLTransformer scheme, which more effectively enhances the identification of allosteric residues while suppressing those of orthosteric residues, owing to the use of an allosteric ligand as the input in this case study. Moreover, this performance may also benefit from the effective integration of ESM2 features relevant to coevolutionary correlation with PLTransformer, which thus enabled us to extract evolutionarily and functionally important residues through a combined contribution of ESM2 and PLTransformer.

As the fourth case study for allosteric site identification, we analyzed phosphatidylinositol 5-phosphate 4-kinase (PI5P4K) (UniProt: Q8TBX8). In 2022, Boffey et al. reported selective PI5P4K inhibitors exhibiting a non-ATP-competitive, allosteric binding mode (ligand: DVF) (PDBID: 7qie).^59,60^ Notably, neither the protein nor the ligand is included in the PDBbind data set (v.2020). The authors identified both ATP site and allosteric site binding modes, which were associated with the conformational changes in the activation loop. As shown in Figure 5A, most residue clusters highlighted by PLTransformer (Figure 5C) are located around the ligand binding sites, including the activation loop (Ile375). Interestingly, a higher contact score was obtained for residues associated with the allosteric binding mode (*S*_contact_ = 0.78) than for those of the ATP binding mode (*S*_contact_ = 0.51).

**Figure 5:**
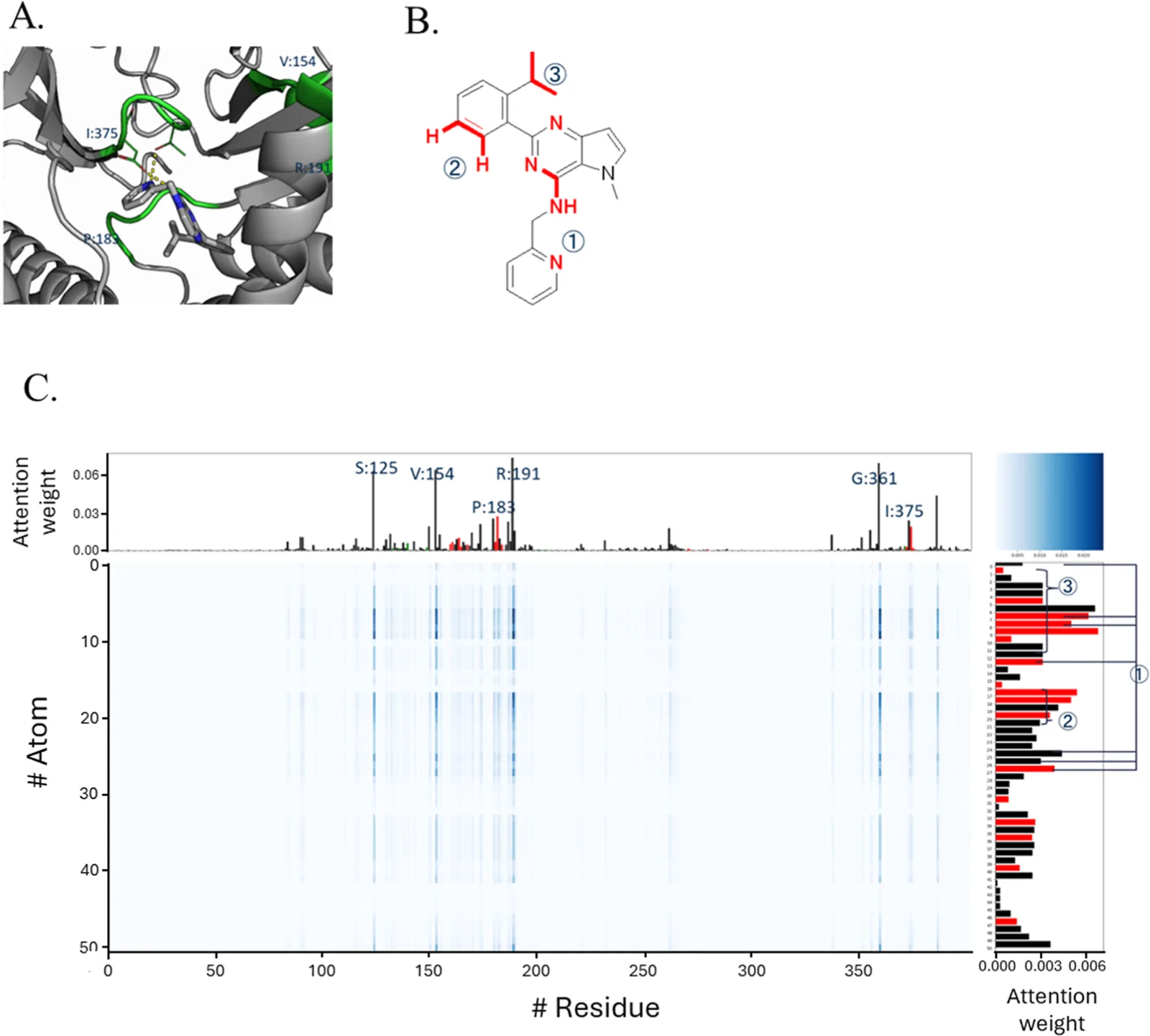
Analysis of phosphatidylinositol 5-phosphate 4-kinase (PI5P4K) (UniProt: Q8TBX8), which is investigated as an unseen allosteric complex (7qie). (**A**) Crystal structure of the PL complex (7qie). Residue clusters identified to be important by PLTransformer are marked in green. (**B**) 2D structure of the ligand in the crystal structure (7qie). Substructures identified to be important by PLTransformer are marked in red. (**C**) The top vertical bar chart displays the sum of all attention weights distributed from each residue to all ligand atoms (*P*_contact_), where the red bars represent the ground-truth residues in contact with the ligand. The horizontal bar chart on the right side shows the sum of all attention weights distributed from each atom to all residues (*D*_contact_), where the regions numbered 1–3 correspond to the numbered substructures in (B), and the red bars represent the ground-truth atoms in contact with the protein. The middle heatmap shows the calculated contact matrix (*M*_contact_), and darker colors indicate higher values.

In terms of the ligand recognition of PI5P4K, our PLTransformer highlighted important atoms in the scaffold predicted to bind to the hinge area (substructure 1 in Figure 5B), the nitrogen atoms of the pyrimidine, and the adjacent aromatic ring (substructures 2 and 3 in Figure 5B). In the best binding mode obtained from a docking simulation, which is consistent with the crystal structure (7qie), the hinge-binding scaffold interacts with Thr377 and Asp374 in the activation loop, and the isopropyl group of the ligand is buried in the pocket, forming contacts with Pro183. Although the docking simulation suggested possible multiple binding modes, our PLTransformer was able to identify a single site in the protein based on the assumption that the hinge-binding scaffold (substructure 1) and 2-isopropylphenyl group (substructure 3) are key contributors to the interaction.

Thus, in this case study, PLTransformer enables us to identify plausible 3D structures of PL complexes, even among multiple binding modes derived from docking simulations. These results suggest that our model has the capability to identify residues associated with allosteric regulation and to propose binding modes that are consistent with ligand atoms identified as being important for PL recognition. Furthermore, integration with relevant methodologies, such as advanced docking^51^ and MD simulations, may further enhance the determination of biologically plausible allosteric binding configurations.

### Serine Protease

As the fifth case study, we built a PLTransformer model with respect to serine protease, whose structures contain a serine residue in their catalytic sites, with the substrate binding pockets conserved around the catalytic center, named S1, S2, S3, and S4 pockets. In this report, we propose a contact score *S*_contact_ as an auxiliary score in the model section to provide a great improvement in the model’s interpretability.

First, we obtained a data set of proteins belonging to the serine proteinase family from a peptidase database, MEROPS.^61^ For affinity data, we gathered positive binders from BindingDB^62^ and negative binders from PubChem.^63^ Thus, the input data contains 86,778 PL pairs (for more detailed statistics, see Table 2). The training statistics of PLTransformer model employing this data set are shown in Table 3 (the affinity values ranged from 2.00 to 11.77 as shown in Table 2). We obtained a PLTransformer model exhibiting a better affinity correlation than that achieved by the model employing PDBbind data set, as shown in Table 3 (for the scatter plot between true and predicted values, see Figure S1 in the Supporting Information). Thus, the presented results suggest that the model specialized for the specific protein family can improve the affinity evaluation. Next, we identified residues important in the ligand recognition; for the evaluation of our scheme, we analyzed human trypsin (UniProt: P07477) and its ligand, for which the crystal structure of the PL complex is reported (PDB ID: 5lgo). As a consequence, W216 in the S4 pocket exhibited the highest protein attention weight (*P*_contact_) value of all residues (Figure 6B). We computed the contribution of each atom in the ligand to W216, which was represented by μ­(W216) (see the Methods section for details). As a result of the analysis, P1 (28C), P2 (13C, 22C), and P4 (16O) groups were found to exhibit high attention weight values against just a single residue W216 (Figure 6C).

**Table 2:** Summary of Training Data Sets

	PDBbind	serine proteinase
No. of unique proteins	2994	292
No. of unique ligands	12,397	57,361
No. of PL pairs (train/validation/test)	16,113 (14,324/1433/356)	86,778 (69,422/8678/8678)
pIC50 range	0.40–15.22	2.00–11.77

**Table 3:** Results of Our Model and Baseline Model (TransformerCPI)

	data set	RMSE	R2
transformercpi	PDBbind	1.41	0.56
our model	PDBbind	1.15	0.62
our model	Serine proteinase	0.89	0.77

Note herein that Jin et al.^48^ previously employed a cross-attention mechanism^49,50^ to capture mutual effects between protein binding pockets and ligands by changing the roles (query, key, and value) of proteins and ligands across different attention blocks. In contrast, our approach fundamentally differs from theirs in architecture: their attention blocks are arranged in parallel, whereas ours are arranged sequentially, enabling denser interactions between the two attention blocks. As a result, our PLTransformer does not require explicit positional information for ligand atoms responsible for PL interactions, while the prior work relied on structural information on protein-binding pockets to extract key residues.

**Figure 6:**
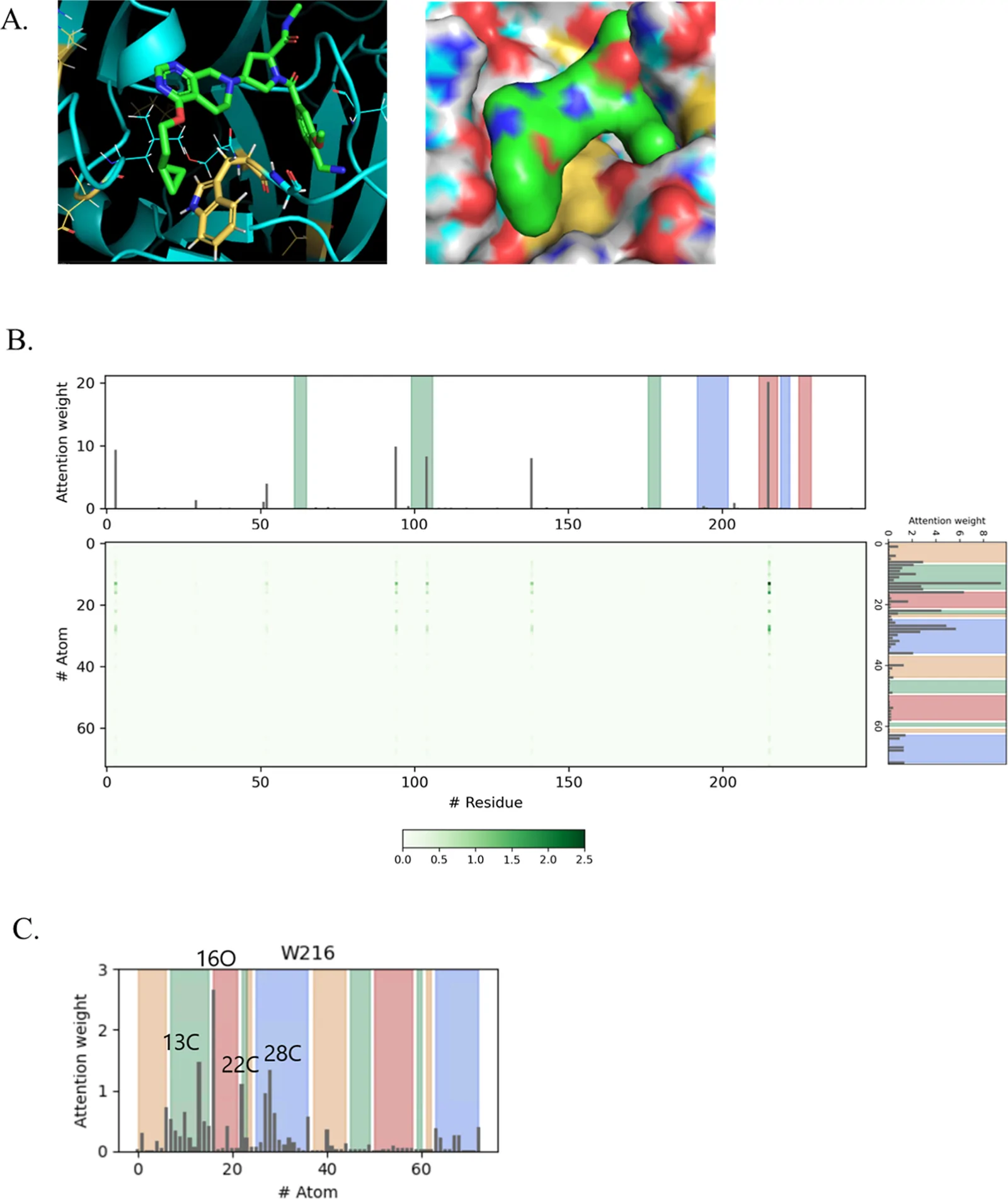
Attention analysis of protein–ligand complex from the serine protease data set. (**A**) 3D structure of trypsin-ligand complex (PDB ID: 5lgo). The left image shows trypsin as a cyan ribbon (Cα) and the ligand as a green (C atom) wire. The right image shows trypsin and the ligand (green) as the molecular surface. The focused residue (W216) is highlighted by yellow. As shown herein, the ligand broadly surrounds W216, thus forming intensive contact with this single protein residue. (**B**) The top vertical bar chart displays the sum of all attention weights distributed from each residue to all ligand atoms (*P*_contact_), where the red bars represent the ground-truth residues in contact with the ligand. The horizontal bar chart on the right side shows the sum of all attention weights distributed from each atom to all residues (*D*_contact_), where the colored regions correspond to the ligand functional groups. The middle heatmap shows the calculated contact matrix (*M*_contact_), and darker colors indicate higher values. (**C**) The attention weights of ligand atoms with respect to W216 (μ­(*W*216)).

Note herein that significant columns in the contact map are apparently similar to each other in terms of their vertical patterns (see the lower panel in Figure 6B); however, this is not actually the case. More specifically, the vertical pattern of the W216 column differs from those of other residues (see Figure S2 in the Supporting Information), which suggests that the contact map represents distinct contributions of ligand atoms to each residue, although spatially adjacent segments in the PL complex are involved in related interactions.

In fact, for W216 in the crystal structure, the aforementioned functional groups of the ligand broadly form hydrophobic interactions with the backbone and/or side chain of this residue (see the left panel in Figure 6A) and thus making an exclusively intensive hydrophobic molecular contact in the PL complex (see the right panel in Figure 6A). This picture, which, to the best of our knowledge, has been described for the first time in this report, can unambiguously provide an explanation of the high PL affinity (6 nM^64^). Actually, the previous experiment using derivatives of this ligand also showed that an extension of functional subgroups from the catalytic center (S1 pocket) to S4 pocket further increased the affinity.^64^ Note herein that PLTransformer thus led to the aforementioned results (showing that ligand atoms identified to be important for contact with W216 are actually critical for binding in the catalytic site of trypsin) in a manner independent of 3D protein structures.

### Use of Known 3D Structures of PL Complexes for LBDD and SBDD

Recently, MONN,^28^ SEGSA_DTA,^29^ and ArkDTA^23^ have explicitly utilized known NCI labels, which are calculated from atomic coordinates of 3D structures of PL complexes, to constrain the distribution of the attention matrix to approach the NCI labels during training. Although attention on both proteins and ligands can be observed through this so-called “symptomatic treatment” approach, which uses known facts and prior knowledge, the training data sets have been limited to PL pairs with known 3D structures. They emphasized that such a fine-tuning scheme was useful for limited data sets that lack 3D structural information. However, great effort is still required to construct pretrained and fine-tuned models.^23,29^ Moreover, given that the pretraining data sets used in these models are quite small (PDBbind <20k), it is not realistic to extend fine tuning to public million-scale data sets (for example, ChEMBL data set^65^). Although MONN and ArkDTA present the pairwise interaction through the AUC score or visualized heatmap of the attention matrix, they did not show a systematic analysis of PL contacts within the context of SBDD.

Unlike the approaches discussed above, the attention weights of our proposed models can identify important ligand atoms as well as protein residues involved in ligand binding, although our modeling scheme is independent of the explicit 3D structural features. We employed TransformerCPI^20^ as a representative baseline model because, like PLTransformer, it does not require 3D structural information for training. Using identical data sets (PDBbind), PLTransformer demonstrated improved predictive performance over the one-side-attention baseline model. Consequently, this comparison corresponds to a one-side versus two-side attention mechanisms and demonstrates the contribution of our proposed attention scheme to affinity prediction task. A broader benchmark against recently developed state-of-the-art PL-affinity prediction models, including ones employing 3D structural data for training and inference, is thus identified as an important direction for our future work. In PLTransformer, we proposed a novel two-side attention mechanism for PL affinity and contact analysis. We normalized the attention matrix both on protein (residue) and ligand (atom) directions step-by-step, thereby resulting in a square attention matrix analogous to that used in the self-attention mechanism. The two-side normalization directly on the attention matrix was realized by a novel, two-step separated methodology. First, Softmax normalization on residue-direction was applied on a rectangular matrix, and the output feature (with the same size as the input ligand feature) together with the ligand feature were fed into the Decoder, which results in an *n* × *n* square attention matrix. Softmax normalization was then applied to the atom’s direction. Moreover, roles of input feature (Q, K, and V in the attention mechanism) were intentionally reassigned between the two decoder blocks. Finally, the resultant, merged feature is aggregated and fed to a neural network for binding affinity prediction. Furthermore, by reconstructing the attention matrices in Encoder and Decoder, we obtain a contact matrix between residues and atoms, which enables us to map PL contacts as residue-atom contacts qualitatively.

Analyzing the attention matrix, we further successfully obtained information concerning PL contact, orthosteric and allosteric binding pockets, and important atoms in ligands. One can use such information to integrate with the subsequent analyses such as docking and/or MD simulations aiming at the hybrid LBDD/SBDD (HLS) work. We also showed that both affinity correlation and contact information, which can be obtained by PLTransformer simultaneously, were actually helpful for the appropriate selection of 3D PL structural models. Notably, the contact maps derived from the attention matrices showed consistency with the interaction patterns observed in experimentally determined crystal structures. Although PLTransformer does not explicitly utilize three-dimensional structural information during training, the model was able to identify residues and ligand functional groups involved in binding and capture the local environment surrounding the binding site. These observations suggest that the attention matrices contain biologically relevant information beyond the affinity evaluation. Furthermore, when multiple docking poses were generated for a given protein–ligand pair, the agreement between the contact maps and structurally plausible interactions provided useful guidance for identifying the most likely binding mode. Therefore, PLTransformer can serve as not only an affinity prediction model but also a complementary tool for structure-based analyses and binding mode assessment. Herein, the use of a single-family data set may be more effective for analysis to clarify the specific binding mode, which is a point of improvement. Thus, the square attention matrix scheme presented herein provides a framework to extend our molecular design analysis toward integration of LBDD with the SBDD approach as an HLS analysis scheme.

## Conclusions

We showed that mitigating the gap between multimodal data spaces such as protein residues and ligand atoms improved the performance of the cross-attention mechanism. We can expand this idea for further improvement: one possible extension is to formulate a tensor representation such as to more systematically integrate multimodal data spaces and then apply an attention mechanism on the tensor space, and another is modifying the doubly stochastic normalization, which is provided in Sinkformer,^22^ to fit our rectangular attention matrix. Thus, our PLTransformer may provide more substantial representations of PL interactions to determine binding modes through the HLS scheme, which thus enables us to unify various obtained data to understand the binding modes and ligand affinities for molecular design.

## Supplementary Material





## Data Availability

The source code is available from Zenodo: 10.5281/zenodo.18307597. The PL-affinity labels and feature vectors of proteins and ligands from the training data are available from Zenodo: 10.5281/zenodo.18307390.

## Notes

Attached are the affinity labels from training data and supporting figures. The Supporting Information is available free of charge at https://pubs.acs.org/doi/10.1021/acsomega.6c04325.

Prediction result of serine protease-specific PLTransformer model and attention analysis of PL complex derived from serine protease dataset (PDF)
PL-affinity tables from PDBbind, BindingDB and PubChem, split into training, and validation and test sets (ZIP)

## NOMENCLATURE

PL: protein–ligand
NCI: noncovalent interaction
NLP: natural language processing
MD: molecular dynamics
SBDD: structure-based drug design
LBDD: ligand-based drug design
RMSE: root-mean-square error
AR: androgen receptor
ABS: androgen binding site
ATP: adenosine triphosphate
HLS: Hybrid LBDD/SBDD.
