# Supplementary material for: A Hybrid Ligand- and Structure-Based Drug Design Invoked through Square Attention in Protein–Ligand Transformer
Source: ACS Omega. 2026 Oct 8;11(44):68439–52. doi: 10.1021/acsomega.6c04325 (PMC13756707; doi:10.1021/acsomega.6c04325)
Supplement: Supplementary file 1 [file ao6c04325_si_001.pdf]

A Hybrid Ligand- and Structure-Based Drug Design Invoked through Square Attention in Protein-Ligand Transformer.

Yanhui Lu, Yasunori Ohara, Hayato Kunugi, and Masaru Tateno*

Innovation to Implementation Laboratories, Central Pharmaceutical Research Institute, Japan Tobacco Inc., Takatsuki, Osaka 569-1125, Japan

Email: tateno0112@gmail.com

KEYWORDS. Protein-Ligand affinity, Transformer, Artificial Intelligence, Structure-based Drug Design

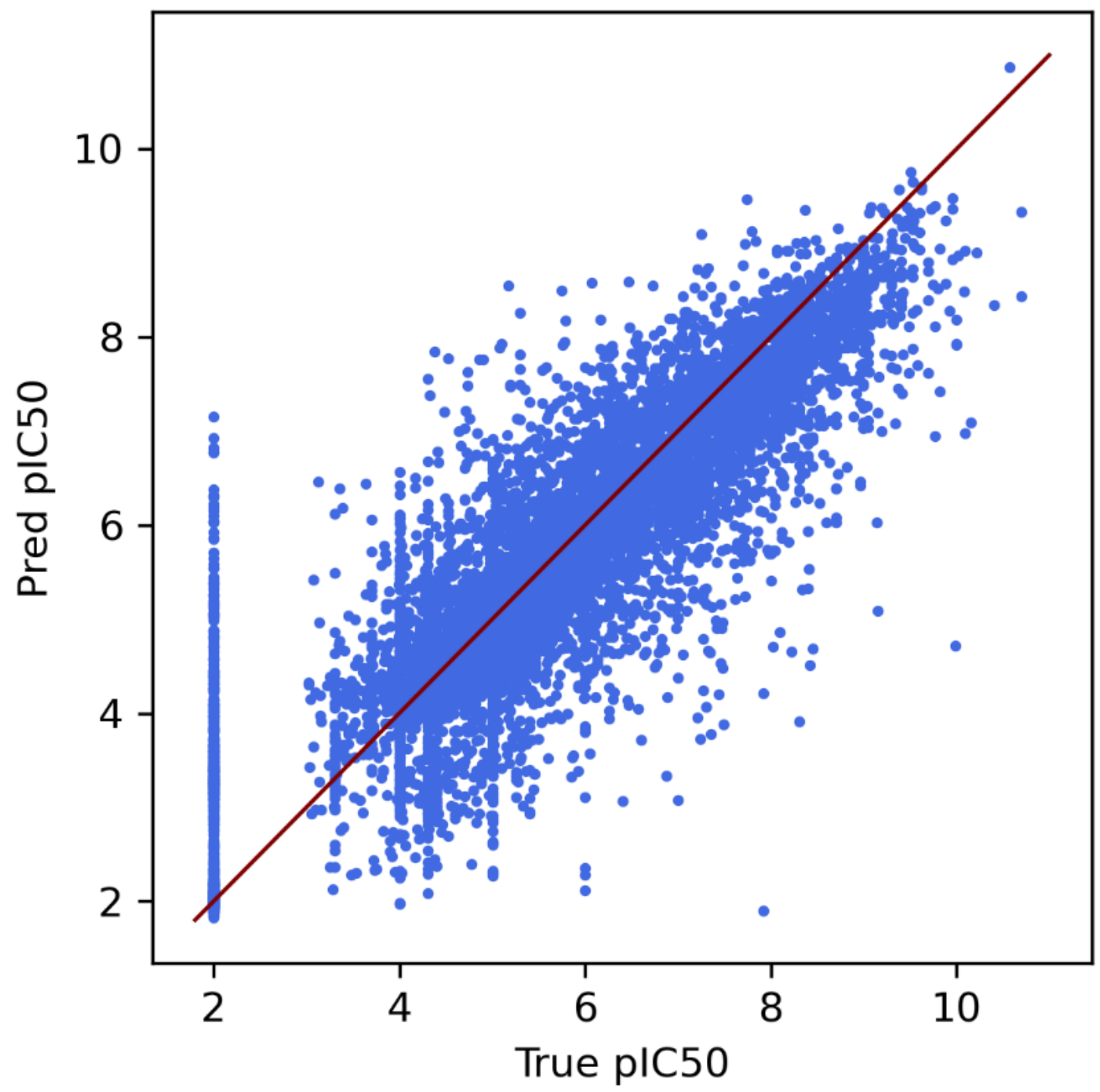

Figure S1. Prediction results of the serine protease-specific PLTransformer model. Blue points represent PL pairs in the test dataset, with true affinity values (pIC50) on the x-axis and values predicted by the model on the y-axis. The red line indicates that the predicted and true values are identical (y = x). Since the affinity values of the negative samples are uniformly assigned to 10 mM (pIC50 = 2.0), the corresponding points are vertically aligned at x = 2.0.

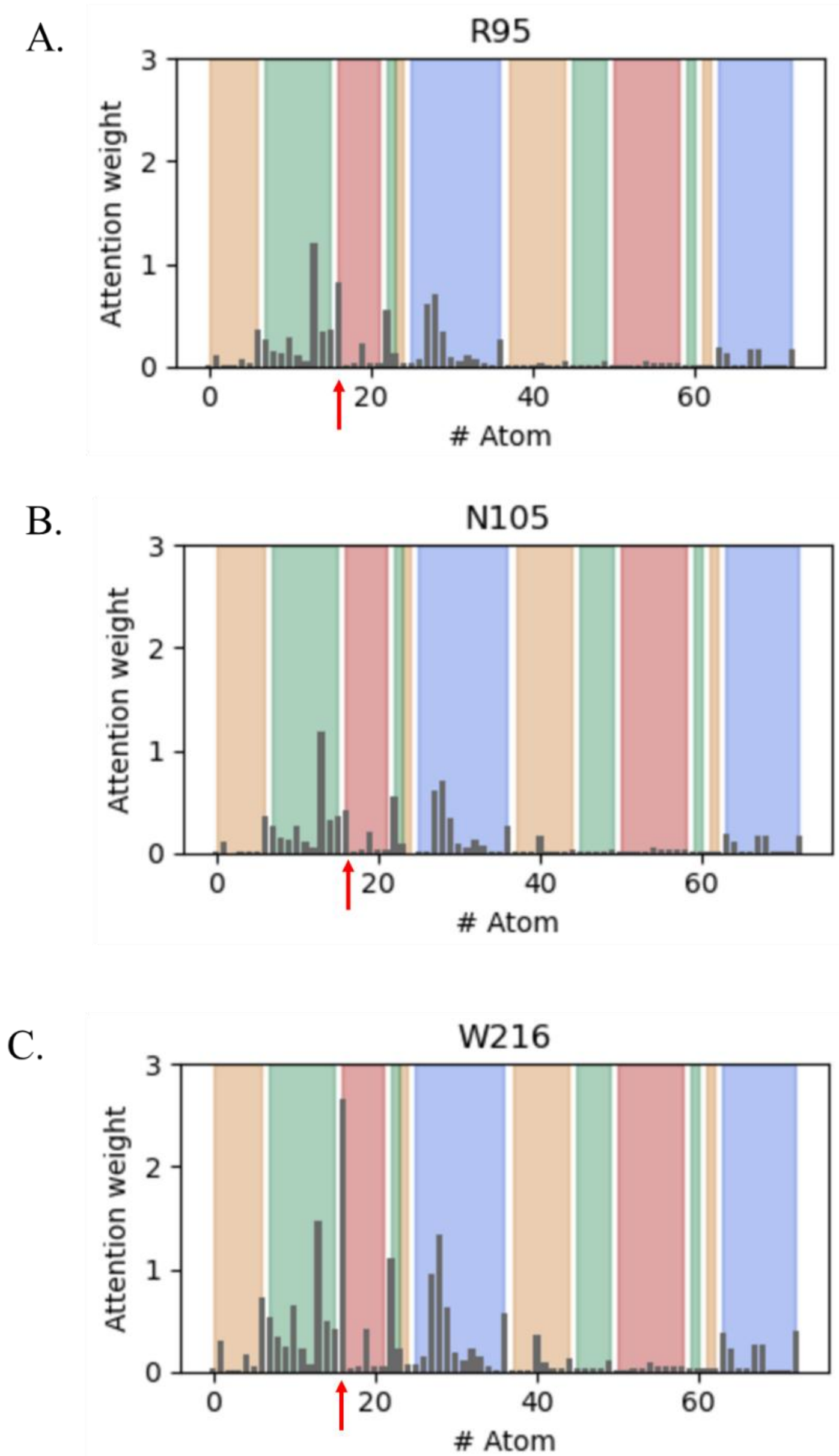

Figure S2. Attention analysis of the PL complex (PDB ID: 5lgo) derived from the serine protease dataset. (A-C) Attention weights of ligand atoms with respect to R95 (μ(R95)), N105 (μ(N105)) and W216 (μ(W216)), respectively. The red arrows indicate the ligand atom with the highest attention weight for W216 (16O). Panels A-C show that the attention weights of this ligand atom differ depending on the residue. From a global perspective, the attention weight patterns across ligand atoms also differ among protein residues.
